# Protein–fragment complex structures derived by NMR molecular replacement†
†Electronic supplementary information (ESI) available: ESI Fig. S1–S8, and Table S1 and S2. See DOI:10.1039/d0md00068j


**DOI:** 10.1039/d0md00068j

**Published:** 2020-04-27

**Authors:** Felix Torres, Dhiman Ghosh, Dean Strotz, Celestine N. Chi, Ben Davis, Julien Orts

**Affiliations:** a Laboratory of Physical Chemistry , ETH , Swiss Federal Institute of Technology , HCI F217, Vladimir-Prelog-Weg 2 , 8093 Zürich , Switzerland . Email: julien.orts@phys.chem.ethz.ch; b Vernalis , Granta Park , Cambridge , UK

## Abstract

Recently we have established an NMR molecular replacement method, which is capable of solving the structure of the interaction site of protein–ligand complexes in a fully automated manner. While the method was successfully applied for ligands with strong and weak binding affinities, including small molecules and peptides, its applicability on ligand fragments remains to be shown. Structures of fragment–protein complexes are more challenging for the method since fragments contain only few protons. Here we show a successful application of the NMR molecular replacement method in solving structures of complexes between three derivatives of a ligand fragment and the protein receptor PIN1. We anticipate that this approach will find a broad application in fragment-based lead discovery.

## Abstract 2

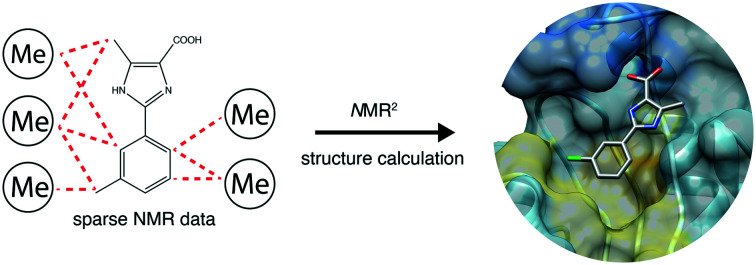
The *N*MR^2^ method can derive protein–fragment structures with a cooperative assignment strategy, opening an avenue for NMR-based fragment lead discovery.

## Introduction

Fragment-based lead discovery (FBLD) is a powerful method used to identify chemical starting scaffolds and optimize them to obtain lead molecules. FBLD provides a clear structure activity relationship with simple chemical building blocks that can be relatively easily functionalized and cover a significant amount of chemical diversity.^1^–^3^ This strategy has been successful against several targets which proved difficult for traditional drug discovery process.^1^ The hits are generally weak affinity binders, and can subsequently be optimized with the use of structural information.^4^ X-ray crystallography is usually the prime method for deriving three-dimensional structures, but this can sometimes be difficult, especially for early fragment hits with low affinity.^5^,^6^

While NMR is a highly versatile structural technique providing structural as well as dynamic information, its application in the context of structure based drug design remains infrequent.^7^ This is mainly due to the laborious process of determining macromolecular three-dimensional structures by NMR. The complexity of spectra acquisition and interpretation as well as considerable time required for resonances assignments, derivation of distance restraints and structure calculations remains a cumbersome and time-consuming procedure compared to the equivalent protocols used in X-ray crystallography. Recently, we introduced the NMR molecular replacement (*N*MR^2^) that enables the fast and robust NMR structure calculation of a protein–ligand interaction site with the use of semi-ambiguous NOE distance restraints.^8^–^11^ *N*MR^2^ structure determination of the complex generally follows a straightforward three steps protocol: (i) preparation of the sample with either the protein or ligand isotopically labelled. (ii) Acquisition of NMR experiments to assign the ligand(s), identify the protein methyl group resonances and derive the intra-ligand and protein–ligand inter-molecular distance restraints. (iii) *N*MR^2^ structure calculation protocol.^8^,^9^ While this is the standard *N*MR^2^ process, the method is versatile and can be tailored to the investigated system. *N*MR^2^ overcomes the barrier of the time-consuming protein assignment step and only requires the interpretation of simple NMR spectra. The method is applicable from hit validation to hit to lead stages, and was recently shown to be suitable for the determination of the structures of protein–ligand complexes with both strong and weak binders.^8^,^9^ However, fragments often contain relatively few observable protons, and thus few intermolecular NOEs can be measured. The small number of intermolecular distance restraints available to derive the structure of a protein–fragment complex is a strong limitation for NMR and in particular *N*MR^2^ since the inter-molecular distance restraints are not assigned on the protein side and therefore remain ambiguous. Here we show how to optimize the *N*MR^2^ approach to derive the structure of a set of fragments in complex with their target, the prolyl isomerase PIN1.

PIN1 is a peptidyl-prolyl *cis*/*trans* isomerase that recognizes phospho-serine/threonine-proline motifs, and a critical modifier of multiple signalling pathways. It is overexpressed in several cancers and its activity contributes to tumour initiation and growth.^12^,^13^ Several studies reported inhibitors of PIN1 but no drug has yet reached the market.^14^,^15^ One of the first developed inhibitor, juglone, did not lead to a drug candidate due to the lack of selectivity.^16^ A large number of phenylimidazole fragments were previously identified as binding to PIN1.^17^

## Results and discussion

**Fig. 1:**
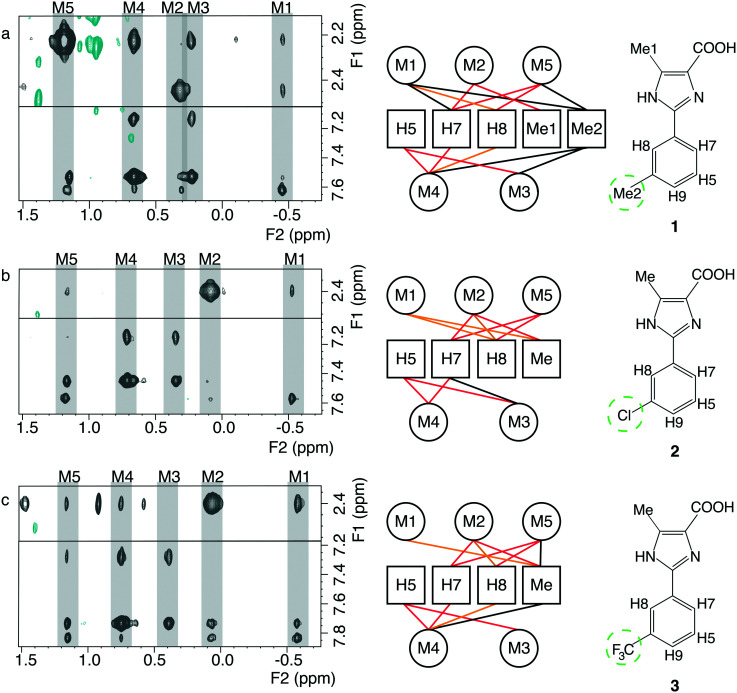
Overview of the NMR restraints for the compounds **1**, **2** and **3**, displayed in panel a–c respectively. Left) Intermolecular cross-peaks from F1-[^15^N,^13^C]-filtered [^1^H,^1^H]-NOESY spectra of PIN1 in complex with the corresponding fragments. The protein methyl groups involved in inter-molecular NOE(s) are arbitrarily named M1 to M5 and the ligand resonance assignments are reported in Table S1.† Middle) Intermolecular distance restraint network between the assigned fragment's protons and the unknown protein's methyl groups. The red lines show the restraints that are shared between all the fragments, the orange lines represent restraints that are shared between two fragments, and the black lines represent restraints that are specific to the considered fragment. Right) Structures of the fragments. The isosteric groups are emphasized by the green dashed circles.

Compounds **1**, **2** and **3** targeting PIN1 share a common phenylimidazole scaffold substituted with the isostere groups CH_3_, Cl, and CF_3_, respectively, Fig. 1.

PIN1 was expressed as doubly [^15^N,^13^C]-labelled protein in order to discriminate between the NMR signal of the protons from the protein and the protons from the fragments. Three NMR samples containing 1.3–1.5 mM PIN1 and 3 mM ligands were prepared for subsequent NMR measurements (see Experimental section). The protein methyl group resonances were identified by collecting ^13^C-ctHSQC spectra for each complex (see below). The fragment assignments were straightforward using 1D ^1^H NMR spectra in the free form and in complex with the PIN1, as well as the F_1_-[^15^N,^13^C]-filtered [^1^H,^1^H]-NOESY experiments (see below), Table S1.†

A series of F_1_-[^15^N,^13^C]-filtered [^1^H,^1^H]-NOESY spectra were recorded on a 900 MHz spectrometer for each PIN1–fragment complex.^18^ A total of 17, 14, and 18 inter-molecular NOEs, could be measured for the fragment **1**, **2**, and **3** respectively. Build-up curves of poor quality or showing quadratic behaviours characteristic of spin diffusion, were discarded. The cross-relaxation rates derived from the NOE build-up curves were converted to distances using the effective correlation time of the complexes. The effective correlation times, defined from the population averaged correlation times of the free and bound fragments, were derived from the apo- and holo-populations calculated from affinity measurements and when possible, from the sterically known distances from the fragments (Table S2†).^19^

The binding affinity constants were derived using protein methyl group chemical shift perturbations upon fragment titration and found to be *K*_D,1_ = 260 μM, *K*_D,2_ = 670 μM, and *K*_D,3_ = 6700 μM (Table S2, Fig. S1–S3†). A set of 14, 11, and 12 distances for the fragment **1**, **2**, and **3** respectively were retained for the *N*MR^2^ structure calculations (see ESI† Fig. S4). The fragment conformations were found to be planar in agreement with the intra-NOE derived distances measured in their bound states (see Experimental section), as well as *ab initio* calculations using the software Gaussian. The *N*MR^2^ calculations were performed at the known catalytic site of PIN1 (Fig. S5†) using the experimentally derived distances restraints, the derived ligand structures and a starting structure of the protein arbitrarily taken from a previously determined crystal structure.^17^ Each side chains dihedral angle of PIN1 could rotate by 20° providing a large degree of flexibility to accommodate the ligand. Additional useful information for the structure determination could be easily determined from the recorded set of NMR spectra. One protein methyl group assignment was readily derived from the ^13^C-ctHSQC, and used in the *N*MR^2^ calculations, since the methionine resonance peaks are negative and only one methionine, M130, is present in the binding site (Fig. S5 and S6†). The alignment of the F_1_-[^15^N,^13^C]-filtered [^1^H,^1^H]-NOESY spectra with the ^13^C-ctHSQC spectrum of each complex enables the identification of which NOE restraints involve the M130 methyl group and the protons of the fragments (Fig. S6 and S7†).

Initially, the *N*MR^2^ structure determination for two out of three PIN1–fragment complexes involving compounds **2** and **3**, failed to converge. The 10 best structures exhibited null target functions expressed as the sum of the squares of residuals, suggesting that the calculations are underdetermined and that several complex structures fulfilled equally well the NOE restraints. On the other hand, fragment **1**, for which 14 inter-molecular distances could be determined, converged and therefore also provided the protein methyl assignments involved in the inter-molecular NOEs. These results suggest that the poor convergence of the structure calculation was due to the paucity of restraints, 11 and 12 inter-molecular distance restraints for the complex involving fragment **2** and **3**, respectively. We therefore investigated the complex PIN1–**2***in silico* with the aim to find what would be the minimal amount of restraints sufficient to calculate an *N*MR^2^ PIN1–fragment structure. The distance restraints were taken from the X-ray crystallography structure of the complex (PDB ; 2XP6) with the visualization program Chimera and randomized by 20% with white noise. The number of restraints used in *N*MR^2^ was decreased incrementally by removing large distances first. We observed that the true positive structures among the 10 best structures, exhibiting the lowest target function, systematically decreased to reach random selection when only 13 distances were used, suggesting that the complex PIN1–**2** cannot be derived with fewer than 13 distance restraints.

It is possible that *N*MR^2^ encounters convergence problem with small fragments due to the lack of sufficient protons and their reduced size. Fragments have a low molecular weight and contain generally few protons. Consequently, the number of protein–ligand inter-molecular NOEs is reduced as well as their interaction surface. However, we found that it was possible to determine structures of the two complexes which did not converge using the methyl assignments from the successfully completed PIN1–**1***N*MR^2^ calculation. The alignment of the NOE patterns from the F_1_-[^15^N,^13^C]-filtered [^1^H,^1^H]-NOESY spectra enable the transfer of protein methyl assignments from the *N*MR^2^ derived PIN1–**1** complex structure to the distance restraints of the other fragments whose complex structures could not be derived. The transfer of assignments is greatly facilitated by following the chemical shift perturbations of the PIN1 methyl groups during the fragment titration, previously recorded to determine their binding affinities.

**Fig. 2:**
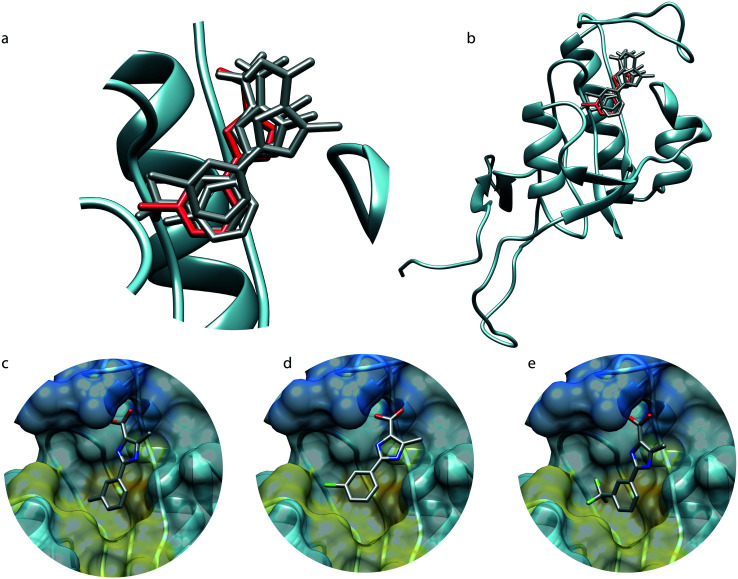
Structure of the PIN1–fragment complexes derived by *N*MR^2^. a and b, Overlap of the *N*MR^2^ structures, depicted in grey, with the X-ray structure of PIN1 in complex with **2** (PDB ; 2XP6), depicted in red sticks and blue ribbon. c–e Complex binding-site structures of **1**, **2** and **3**, respectively, with PIN1 derived by *N*MR^2^. The dark blue surface represents a positively charged region (R68, R69, K63), the yellow colour shows a hydrophobic region (M130, F134, L122), and the gold part, located between the yellow and the blue regions, corresponds to two embedded histidines (H157, H59).

The newly calculated and converged structures are overlaid with the X-ray crystal structure of PIN1–**2** on Fig. 2, and exhibit similar poses to the crystallographic reference with RMSDs of 1.1 Å, 2.5 Å, and 1.4 Å for the fragments **1**, **2**, and **3** respectively. The relatively high RMSD for **2** is due to a slight translation of the fragment toward the outside of the binding pocket. The high similarity in the fragment binding modes to PIN1 could be anticipated since a common NOE pattern can be identified for the three fragments suggesting beforehand a similar binding pose, Fig. 1. Furthermore, the fragments **1**, **2** and **3** are structurally very similar and only differ by an isostere substitution, therefore the binding mode is expected to remain similar. The substituted phenyl group makes hydrophobic interactions with L122, M130, F134, T152, while the embedded histidines, H59 and H157, are candidates for possible π–π or cation–π interactions. The carboxylic acid is involved in salt bridges with the cationic pocket formed by R68, R69 and K63. The imidazole moiety makes hydrogen bonds to S154 and C113, while the trifluoromethyl groups may engage in hydrogen bonding to Q131 and T152.^20^ However steric factors may be the primary reason for the decrease of affinity with CF_3_ (*K*_D_ = 6700 μM, Van der Waals radius ∼ 2.5 Å), compared to CH_3_ (*K*_D_ = 260 μM, radius ∼ 2.0 Å) and Cl (*K*_D_ = 670 μM, radius ∼ 1.8 Å).^21^ Overall the three *N*MR^2^ structures exhibit similar binding modes and are consistent with the reported structure derived by X-ray crystallography.

## Conclusion

Fragment-based drug discovery is a widely used approach in both pharmaceutical companies and academic laboratories. Fragment-based methods need significantly fewer compounds to be screened, synthesized and the fragment hits show usually high ligand efficiencies. Yet fragments contain usually only few protons, rendering the structure elucidation by NMR more difficult. We have shown that the cooperativity between several *N*MR^2^ datasets can overcome the experimental ambiguity due the lack of distance restraints that would otherwise prevent structure elucidation and consequently the corresponding structure-based drug design. The *N*MR^2^ structure calculations only took a couple of minutes of computer time and only a few hours were needed to process and analyse the NMR data so that the complete process perfectly matches the time line required for medicinal chemistry. The time-consuming part of the structure elucidation is no longer the analysis of the NMR spectra series, such as the protein resonances assignment, but rather on the acquisition of NMR experiments. The presented *N*MR^2^ approach may open a new avenue in NMR drug design by enabling fast and robust fragment structure-based drug discovery. Having access to the structure of the binding site for each (weak) binder allows investigating chemical scaffolds that would otherwise be discarded, which broadens the chemical knowledge as well as enables the druggability of the protein targets.

## Experimental section

### Protein expression, compounds and NMR samples

[^15^N,^13^C]-Labelled recombinant PIN1 was produced as previously reported.^22^ PIN1 was buffer exchanged to 100% D_2_O, phosphate 20 mM, sodium chloride 50 mM at pH 6.6 and concentrated to 1.3 mM for **1** and **3**, and to 1.5 mM for **2**. The ligand concentration in the samples used for the NOESY experiments were 2.5, 3, and 3.5 mM for **1**, **2**, and **3**, respectively. Compounds **1**, **2**, and **3** were ordered from Enamine Ltd.

### NMR experiments

All NMR experiments were recorded at 298 K. F_1_-[^13^C,^15^N]-Filtered [^1^H,^1^H]-NOESY spectra were measured with [^13^C,^15^N]-labelled PIN1 and unlabelled ligands on a Bruker Avance 900 MHz spectrometer with cryoprobe, with 2048 complex points in the direct time dimension (*t*_2_) and 241, 256, and 225 complex points in the indirect dimension (*t*_1_) for **1**, **2**, and **3** respectively. The interscan delay was set to 1.5 s, the scans per increment was 160, *t*_2,max_ = 190.05 ms, and *t*_1,max_ = 22.44 ms for **1**, 23.75 ms for **2**, and 20.87 ms for **3**. The mixing times used for the NOE build-ups of **1** were 30, 70 ms, **2**: 40, 60, 90, 120 ms, and **3**: 30, 60, 90 ms. Constant time ^13^C-HSQC (^13^C-ctHSQC) for titration, *T*_1ρ_ and *T*_1_^15^N-HSQC for relaxation experiment and F_1_-[^13^C,^15^N]-filtered [^1^H,^1^H]-TOCSY for fragment proton assignments were recorded on 600 MHz Bruker Avance spectrometer. PIN1 ^15^N-*T*_1_ and ^15^N-*T*_1ρ_ experiments were recorded at 0, 20, 40, 60, 80, 100, 120, 140, 180, 200 ms and 5, 10, 20, 30, 40, 50, 70, 90, 100, 120 ms relaxation delays, respectively.^23^ 64 (*t*_1_) × 2048 (*t*_2_) complex points were used with an interscan delay of 3 s (2 s for *T*_1_), 32 scans per increment, *t*_1,max_ = 138.35 ms, and *t*_2,max_ = 121.65 ms (243 ms for *T*_1_). Spectra were processed with NMRPipe.^24^ For the assignment of the fragments, filtered F_1_-[^13^C,^15^N]-filtered [^1^H,^1^H]-TOCSY spectra were recorded with 1024(*t*_1_) × 2048(*t*_2_) complex points, an interscan delay of 1.2 s, 128 scans per increment, *t*_1,max_ = 13.30 ms, and *t*_2,max_ = 121.65 ms. For the titration of **2** and **3**, ^13^C-ctHSQC spectra were recorded with 256(*t*_1_) × 2048(*t*_2_) complex points, an interscan delay of 1.2 s, 8 scans per increment, a *t*_1,max_ = 71.08 ms, and a *t*_2,max_ = 141.99 ms. The fragment concentrations used for the titration were 60, 156, 252, 348, 444, 540, 636, 732, 828, 924, 1020, 1116, 1308, 1444 μM for **2** and 296, 592, 988, 1183, 1479, 1775, 2071, 2367, 2663, 2959, 3255, 3551, 3847, 4143, 4439, 4735, 5024, 5320, 5616 μM for **3**.

### Data analysis and structure calculations

The effective correlation time of each complex was derived using the population averaged correlation times with *τ*_c,PIN1_ = 8.9 ns and *τ*_c,fragments_ = 0.1 ns. The PIN1 correlation time was calculated using the software Tensor2 and the relaxation rates of the amide groups.^25^ The effective correlation time of each complex was validated using sterically known distances and ligand populations derived from binding affinity constants (see ESI†) and subsequently used to derived the NOE distance restraints. The spectra were processed with topspin 3.1 (Bruker) and analysed with ccpNMR Analysis 2.3.

Distances were computed from NOE build-up curves, assuming an isolated two spin system. First the ligand protons diagonal peaks were fitted to obtain auto-relaxation rates, *ρ*_i_, with a mono-exponential decay Δ*M*_ii_(*t*) = Δ*M*_ii_(0)e^–*ρ*,*t*^. The auto-relaxation rates of the protein methyl groups were assumed to be 4 Hz. The cross-peaks intensities, Δ*M*_ij_(*t*), were normalized to the ligand protons' diagonal peak intensities, Δ*M*_ii_(*t*). The cross-relaxation rate, *σ*_ij_, was then computed from these normalized intensities, 
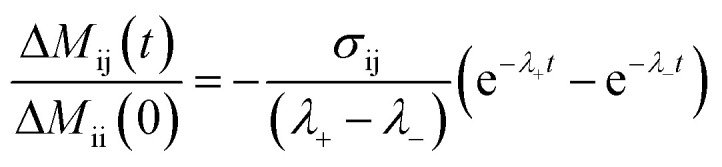
, where 
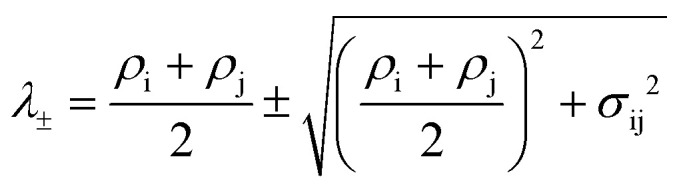
. The auto-relaxation rates of the ligand's protons were obtained from the fitted decay plots of the diagonal peaks. The distances, *r*_ij_, could be extracted from the cross-relaxation rates from of the equation, 
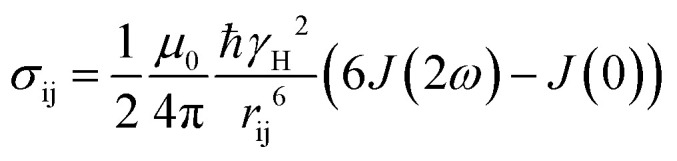
 where 
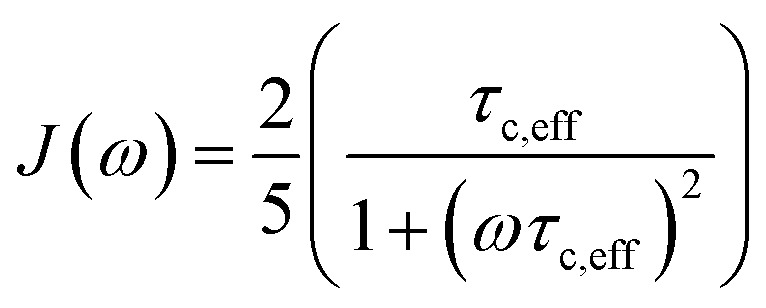
 is the spectral density, *μ*_0_ is the permeability of the vacuum, *ℏ* the reduced Planck constant, *γ*_H_ is the proton gyromagnetic ratio, and *τ*_c,eff_ is the effective correlation time of the protein–ligand complex (Table S2†).

The *N*MR^2^ structure calculations were performed according to the published protocol for the complex PIN1–**1**, and the complexes PIN1–**2** and PIN1–**3** were calculated knowing the protein methyl group assignments.^8^,^9^,^11^ The methyl group assignments determined for the PIN1–**1** complex were cross-validated using a 3D HCCH-TOCSY experiment (Fig. S8†). The fragment geometries were kept fixed during the calculation and optimized in a two steps process: first a molecular mechanics energy minimization was run with Avogadro (UFF force field, steepest descent algorithm); then an *ab initio* energy minimization was run with Gaussian (6-31G(d,p) basis set, DFT B3LYP method). Frequency calculations were run afterwards and the hessians were all positives.

## Conflicts of interest

There are no conflicts to declare.

## Supplementary Material

Supplementary information
