# Supplementary material for: Protein–fragment complex structures derived by NMR molecular replacement
Source: RSC Med Chem. 2020 Apr 27;11(5):591–6. doi: 10.1039/d0md00068j (PMC7649837; doi:10.1039/d0md00068j)
Supplement: Supplementary file 1 [file MD-011-D0MD00068J-s001.pdf]

Electronic Supplementary Material (ESI) for RSC Medicinal Chemistry.
This journal is © The Royal Society of Chemistry 2020

Supplementary Information

Protein—fragment complex structures derived by NMR Molecular Replacement

Felix Torres,[a] Dhiman Ghosh,[a] Dean Strotz,[a] Celestine N. Chi,[b] Ben Davis,[c] and Julien Orts[a]*

[a] ETH, Swiss Federal Institute of Technology, Laboratory of Physical Chemistry, HCI F217, Vladimir-Prelog-Weg 2, 8093 Zürich, Switzerland

[b] Department of medical biochemistry and microbiology, bmc, uppsala University, Sweden

[c] Vernalis, Granta Park, Cambridge, United Kingdom

Corresponding Author *E-mail julien.orts@phys.chem.ethz.ch

Table S1. The ^{1}H NMR chemical shifts of fragments **1**, **2**, and **3**.

Fragment	δ of ^{1}H [ppm]
**1**	2.23 (Me2), 2.45 (Me1), 7.10 (H5), 7.78 (H7), 7.85 (H8)
**2**	2.40 (Me), 7.15 (H5), 7.45 (H7), 7.58 (H8)
**3**	2.40 (Me), 7.25 (H5), 7.75 (H7), 7.85 (H8)

`

Table S2. Affinity, K_D, and effective correlation time, τ_c, of the fragment **1**, **2**, and **3**.

Fragment	Method	K_D [μM]	$\tau_{c,eff}$ [ns]
**1**	Steric distances	260	4.8
**2**	Titration	760	2.4
**3**	Titration	6700	1.0

The effective correlation times of the PIN1–fragment complexes were derived using NOEs from steric distances from the fragment or, when the amount of distances was insufficient, from titration experiments.

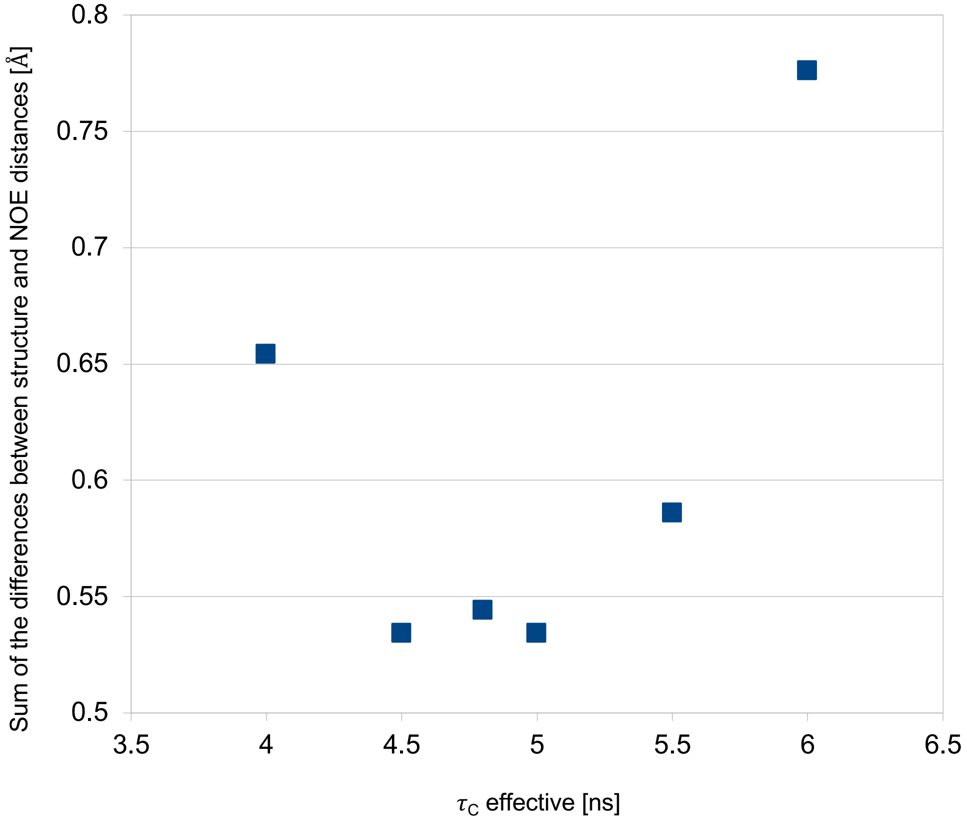

Figure S1. Estimation of the effective correlation time of PIN1—**1**. On the y-axis are reported the sum of the residuals between the steric distances and the experimentally derived distances using the effective correlation time, depicted on the x-axis.

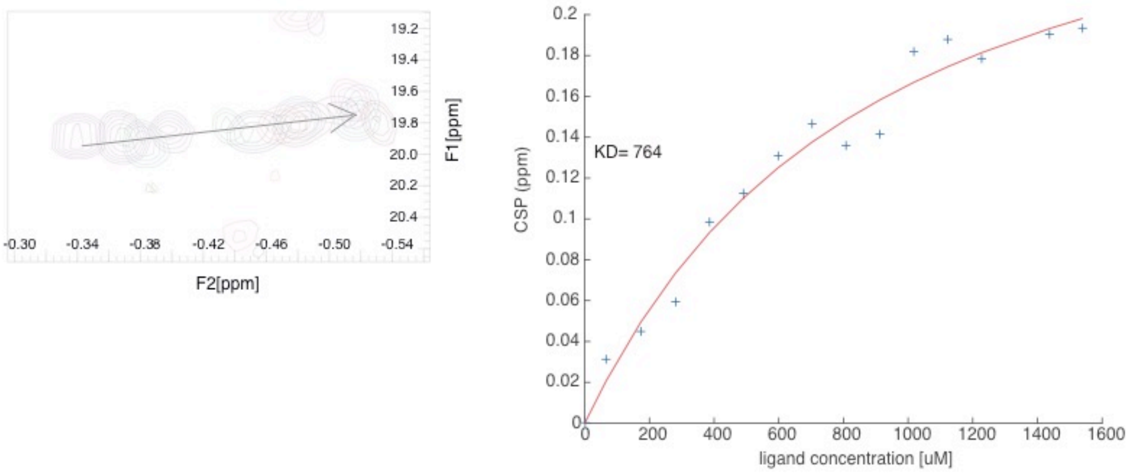

Figure S2. Left) Chemical shift perturbations of the protein methyl group M1 along the fragment **2** titration. Right) The corresponding titration curve used to fit the fragment affinity, K_D.

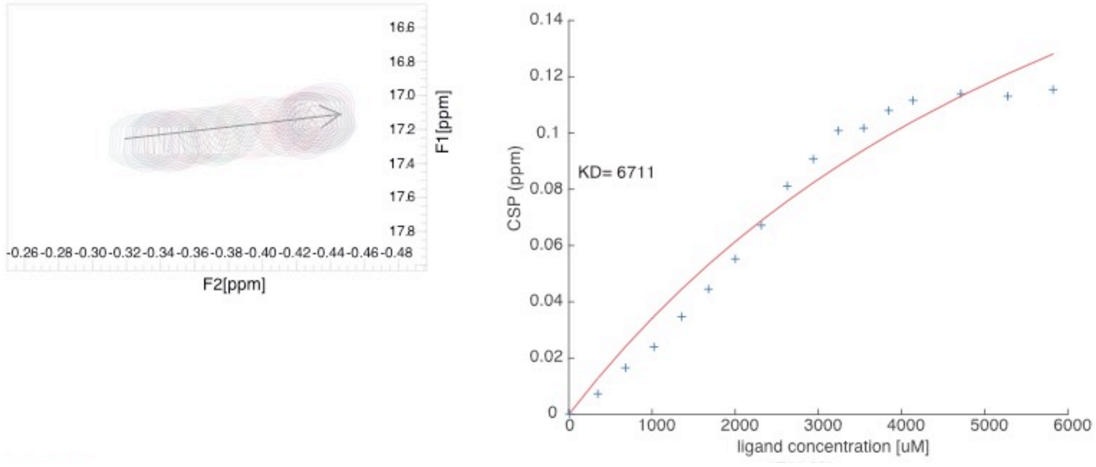

Figure S3. Left) Chemical shift perturbations of the protein methyl group M1 along the fragment **2** titration. Right) The corresponding titration curve used to fit the fragment affinity, K_D.

Compound 1

Protein	Ligand	Distance (Å)
M1	Me2	3.7-6.5
M1	H7	4.6-6.8
M1	H8	3.5-5.3
M2	H7	4.3-6.5
M2	Me1	2.8-5.0
M3	H5	2.3-3.5
M3	Me2	3.5-6.2
M4	Me2	2.8-4.9
M4	H5	2.1-3.2
M4	H7	2.9-4.3
M4	H8	3.9-5.8
M5	H7	3.5-5.2
M5	H8	3.7-5.6
M5	Me2	2.2-3.8

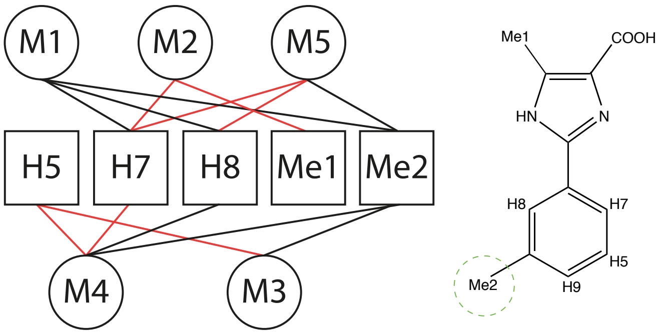

compound 2

Protein	Ligand	Distance (Å)
M1	H8	3.7-6.7
M1	Me	3.5-7.5
M2	H7	4.6-8.2
M2	H8	4.4-7.7
M2	Me	3.1-6.5
M3	H5	2.4-4.2
M3	H7	3.7-6.5
M4	H5	2.3-4.0
M4	H7	3.0-5.3
M5	H7	3.6-6.3
M5	H8	3.6-6.5

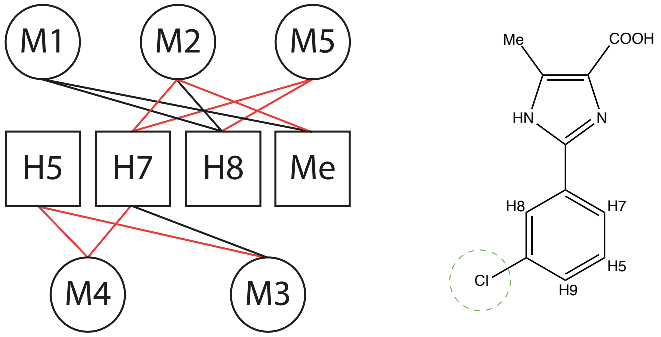

Compound 3

Protein	Ligand	Distance (Å)
M1	Me	2.6-5.5
M2	H7	3.3-5.8
M2	H8	3.6-6.4
M2	Me	2.4-5.1
M3	H5	1.7-3.1
M4	Me	2.8-5.9
M4	H5	1.7-3.0
M4	H7	3.1-4.4
M4	H8	3.6-6.4
M5	H7	3.0-5.3
M5	H8	3.2-5.8
M5	Me	2.8-6.0

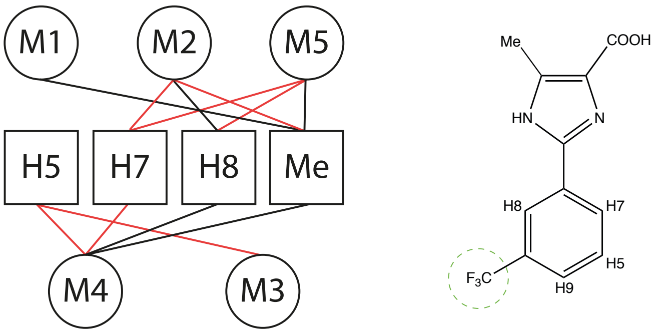

Figure S4. Left) Distance restraint lists for the compound **1**, **2**, and **3**. Middle) Respective distance restraint network. The red lines show the restraints that are shared between all fragments. Right) Structure of the fragment **1**, **2**, and **3**.

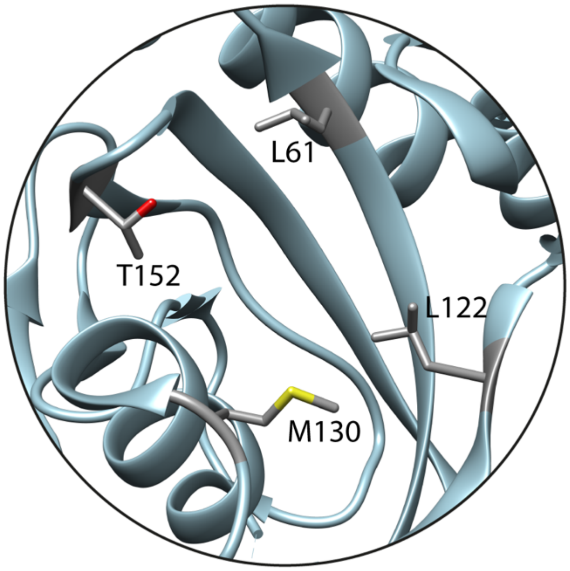

Figure S5. Catalytic site of the protein PIN1. The residues containing methyl groups are depicted with sticks.

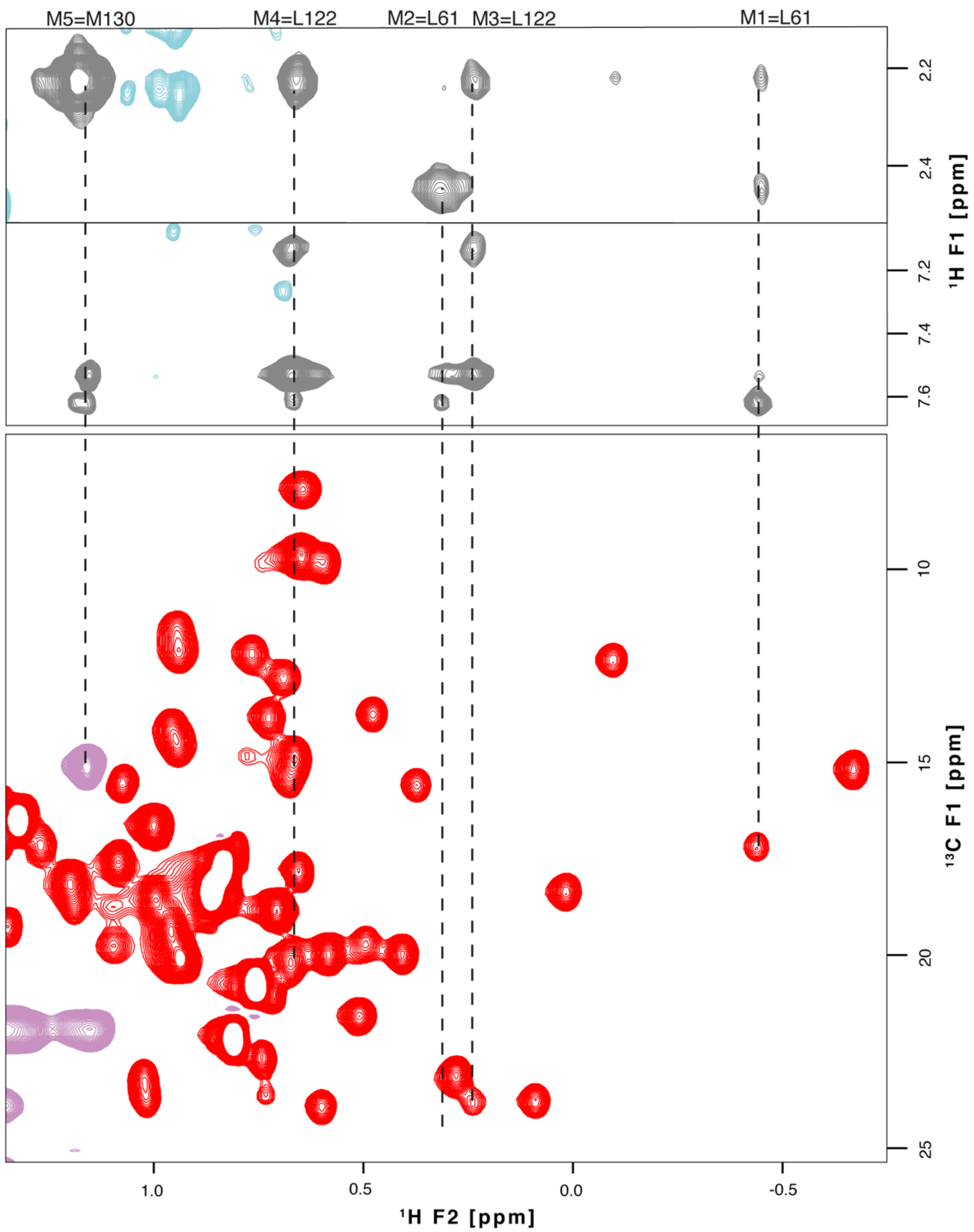

Figure S6. Alignment of the NOESY spectrum (upper part) with ^{13}C-ctHSQC spectra of the same sample (lower part) for the complex PIN1—**1**. All methyl groups could be identified in the ^{13}C-ctHSQC except the methyl 2 (M2) that is below noise level. The absence of the M2 signal was observed for all PIN1—fragment complexes. We could validate the presence of M2 using titration experiments where the M2 proton resonance signal is visible at low fragment concentrations.

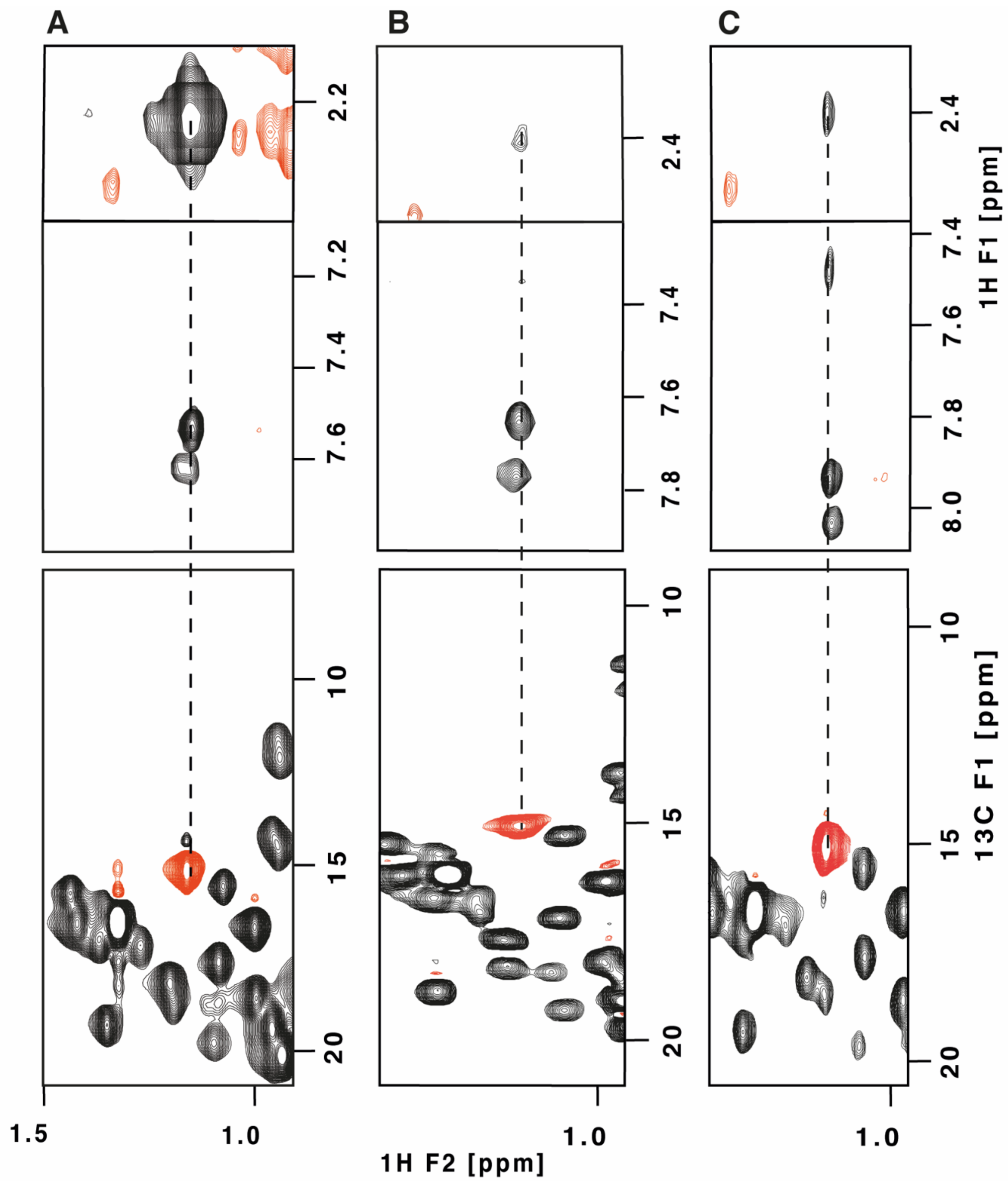

Figure S7. A, B, C Alignment of NOESY spectra (upper part) with ^{13}C-ctHSQC (lower part) of the PIN1—fragment complexes **2**, **3**, and **1**, respectively. The dashed line points to the methionine resonance signal. Negative intensities are depicted in red.

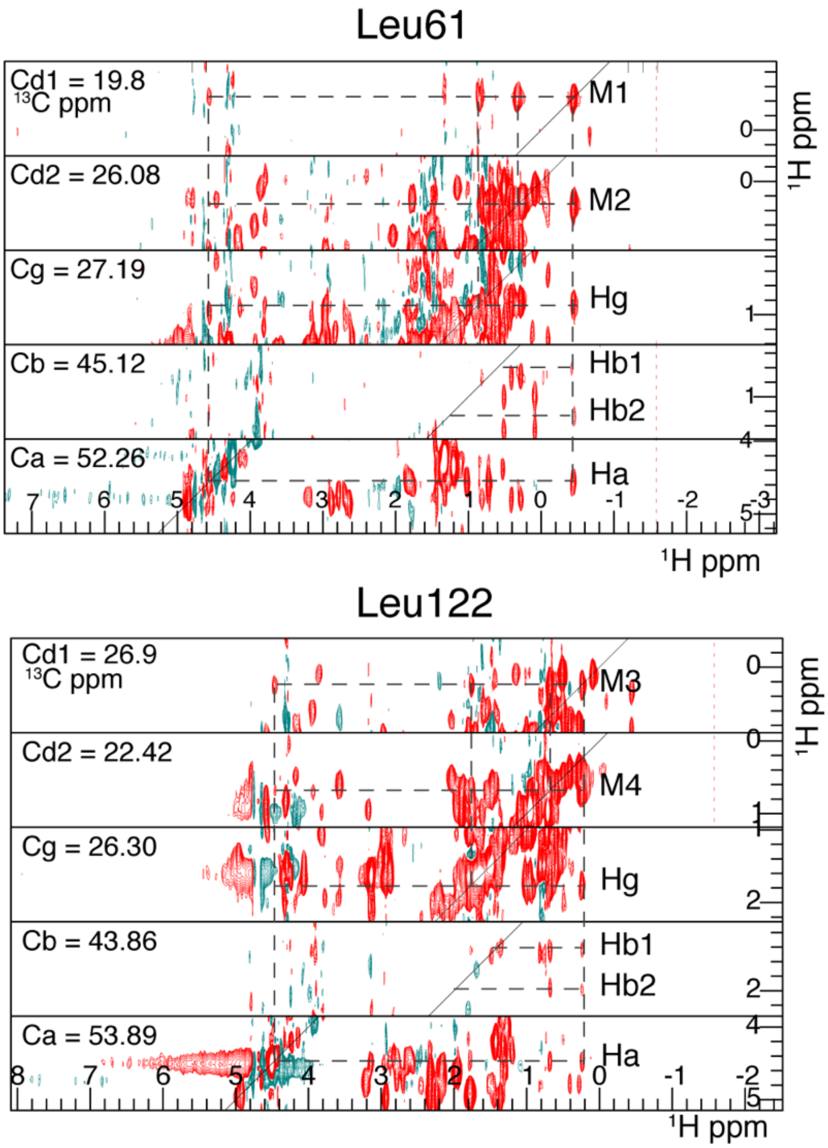

Figure S8. Cross validation of the leucine methyl resonance assignments found by *N*MR^2 using HCCH-TOCSY ^{1}H-^{1}H planes of the PIN1—**1** complex. The methyl groups resonances M1, M2, M3, and M4 belongs to leucine amino acid residues as shown by the 'sequential walk' through the HCCH-TOCSY planes.

1. I. Landrieu, J. M. Wieruszeski, R. Wintjens, D. Inze and G. Lippens, *J Mol Biol*, 2002, **320**, 321-332.
2. N. A. Lakomek, J. F. Ying and A. Bax, *J Biomol Nmr*, 2012, **53**, 209-221.
3. F. Delaglio, S. Grzesiek, G. W. Vuister, G. Zhu, J. Pfeifer and A. Bax, *J Biomol Nmr*, 1995, **6**, 277-293.
4. F. Cordier, M. Caffrey, B. Brutscher, M. A. Cusanovich, D. Marion and M. Blackledge, *J Mol Biol*, 1998, **281**, 341-361.
5. J. Orts, M. A. Walti, M. Marsh, L. Vera, A. D. Gossert, P. Guntert and R. Riek, *Journal of the American Chemical Society*, 2016, **138**, 4393-4400.
6. M. A. Walti, R. Riek and J. Orts, *Angew Chem Int Edit*, 2017, **56**, 5208-5211.
7. M. Wälti and J. Orts, *Magnetochemistry*, 2018, **4**, 12.
